# A Bayesian Ensemble Approach for Epidemiological Projections

**DOI:** 10.1371/journal.pcbi.1004187

**Published:** 2015-04-30

**Authors:** Tom Lindström, Michael Tildesley, Colleen Webb

**Affiliations:** 1 Department of Physics, Chemistry and Biology, Linköping University, Linköping, Sweden; 2 Department of Biology, Colorado State University, Fort Collins, Colorado, United States of America; 3 US National Institute of Health, Bethesda, Maryland, United States of America; 4 University of Exeter, Exeter, United Kingdom; 5 School of Veterinary Medicine and Science, University of Nottingham, Leicestershire, United Kingdom; University of California San Diego, UNITED STATES

## Abstract

Mathematical models are powerful tools for epidemiology and can be used to compare control actions. However, different models and model parameterizations may provide different prediction of outcomes. In other fields of research, ensemble modeling has been used to combine multiple projections. We explore the possibility of applying such methods to epidemiology by adapting Bayesian techniques developed for climate forecasting. We exemplify the implementation with single model ensembles based on different parameterizations of the Warwick model run for the 2001 United Kingdom foot and mouth disease outbreak and compare the efficacy of different control actions. This allows us to investigate the effect that discrepancy among projections based on different modeling assumptions has on the ensemble prediction. A sensitivity analysis showed that the choice of prior can have a pronounced effect on the posterior estimates of quantities of interest, in particular for ensembles with large discrepancy among projections. However, by using a hierarchical extension of the method we show that prior sensitivity can be circumvented. We further extend the method to include a priori beliefs about different modeling assumptions and demonstrate that the effect of this can have different consequences depending on the discrepancy among projections. We propose that the method is a promising analytical tool for ensemble modeling of disease outbreaks.

## Author Summary

Policy decisions in response to emergent disease outbreaks use simulation models to inform the efficiency of different control actions. However, different projections may be made, depending on the choice of models and parameterizations. Ensemble modeling offers the ability to combine multiple projections and has been used successfully within other fields of research. A central issue in ensemble modeling is how to weight the projections when they are combined. For this purpose, we here adapt and extend a weighting method used in climate forecasting such that it can be used for epidemiological considerations. We investigate how the method performs by applying it to ensembles of projections for the UK foot and mouth disease outbreak in UK, 2001. We conclude that the method is a promising analytical tool for ensemble modeling of disease outbreaks.

## Introduction

Epidemiological forecasting is inherently challenging because the outcome often depends on largely unpredictable characteristics of hosts and pathogens as well as contact structure and pathways that mediate transmission [1]. Faced with such uncertainty, policy makers must still make decisions with high stakes, both in terms of health and economics. For instance, global annual malaria mortality was recently estimated at around 1.1 million [2] and to optimize control efforts, policy makers must make seasonal predictions about spatiotemporal patterns [3]. The prospect of an emergent pandemic influenza outbreak remains a global threat and emergency preparedness must evaluate the costs and benefits of control measures such as border control, closing of workplaces and/or schools as well as different vaccination strategies [4]. Livestock diseases are major concerns for both animal welfare and economics. As an example, the United Kingdom (UK) 2001 outbreak of foot and mouth disease (FMD) involved culling of approximately 7 million animals, either in an effort to control the disease or for welfare reasons, and the total cost has been estimated at £8 billion [5]. To minimize the size and duration of future outbreaks, various strategies for culling and vaccination must be compared [6–8]. As a tool to address these challenging tasks, mathematical models offer the possibility to explore different scenarios, thereby informing emergency preparedness and response to epidemics [1,9–12].

The predictive focus of epidemiological models can either be classified as forecasting or projecting [13]. Forecasting aims at estimating what will happen and can be used for example to predict seasonal peaks of outbreaks [3,14] or to identify geographical areas of particular concern [15]. Projecting, which is the main focus of this study, instead aims at comparing different scenarios and exploring what would happen under various assumptions of transmission, e.g. comparing the effectiveness of different control actions [7,16–19].

Whilst analytical models clearly provide important insight into observed dynamics and a theoretical understanding of epidemiology [20–22], there has been a shift in recent years towards stochastic simulation models for predictive purposes [1]. Typically, dynamic models are constructed and outbreaks are simulated repeatedly, thus generating predictive distributions of outcomes [1,17,18,23]. This variability in outcomes caused by the mere stochasticity of the transmission process includes one level of uncertainty, but still only relies on a single set of assumptions about the underlying disease transmission process. However, multiple assumptions can often be justified, leading to further uncertainty in the predictions. For instance, different models may have different projections because of different assumptions about transmission or because they incorporate different levels of detail. It may also be informative to explore different projections in terms of different parameterizations of a single model, for example corresponding to worst or best case scenarios. Faced with a set of projections, an important issue is how to combine these in a manner such that they can be used to inform policy.

The issue of multiple projections is not unique to the field of epidemiology, and various techniques of ensemble modeling have been used to merge projections based on different modeling assumptions. The key concept is that rather than relying on a single set of assumptions, a range of projections is used for predictive purposes. For instance, climate forecasting has employed ensemble techniques to account for uncertainty about initial conditions, parameter values and structure of the model design when predicting climate change [24,25]. Weather forecasting has been improved by combining the results of multiple models [26,27]. Similarly, hydrological model ensembles have been demonstrated to increase reliability of catchment forecasting [28] and have been used to assess the risk of flooding events [29]. Ensemble methods have also proven to be a powerful decision tool for medical diagnostics [30,31] and ecological considerations including management [32] and prediction of future species distribution [33].

Ensemble modeling has not yet been extensively used in epidemiology. However a few implementations exist, commonly by feeding climate or weather ensembles into disease models. Daszak et al. [34] coupled a set of climate projections to an environmental niche model of Nipah virus to predict future range distribution of the virus under climate change. Similarly, Guis et al. [35] investigated the effect of climate change on Bluetongue emergence in Europe by simulating outbreaks under different climate change scenarios. Focusing on a shorter time scale, Thomson et al. [3] used an ensemble of seasonal forecasts to predict the spatiotemporal pattern of within seasonal variation in malaria incidence. These studies all used a single disease model projection, coupled to an ensemble of climate or weather forecasts and the use of structurally different epidemiological models are to our knowledge still rare. However, Smith et al. [36] compared different malaria vaccination strategies by implementing an ensemble approach with different alterations of a base model. Also, in order to estimate global malaria mortality, Murray et al. [2] used weighted averages of different predictive models.

Given the success of ensemble methods in other fields, we expect that epidemiological implementations will increase. For that purpose however, there is a need for methods that combine multiple projections. A central issue in ensemble modeling is how to weight different projections, and we envisage four main procedures for this. Firstly, all models can be given equal weights. For instance, the IPCC 2001 report on climate change [37] used a set of climate models and gave the range of probable scenarios by averaging over different models and uncertainty by envelopes that included all scenarios. Gårdmark et al. [32] used seven ecological models for cod stock and argued that in order to prevent underestimation of uncertainty, weighted model averages are not to be used and when communicating with policy makers, it is preferable to present all included projections as well as the underlying assumptions behind them. A similar approach was also used by Smith et al. [36], who presented the prevalence of malaria under different vaccination strategies by medians of individual models and the range of the whole ensemble.

Secondly, expert opinions can be used to weight models. To our knowledge, no ensemble study has implemented weights based exclusively on expert opinion, but Bayesian model averaging can incorporate expert opinion as a subjective prior on model probabilities [38]. This approach relies on engaging stakeholders and communicating the underlying assumptions of the projections.

Thirdly, models can be weighted by agreement with other models. This approach was implemented by Räisänen and Palmer [39], who used cross-validation to weight climate models. As a more informal approach to the use of model consensus, the third IPCC report excluded two models because these predicted much higher global warming than the rest of the ensemble, thus acting as outliers [24].

Fourthly, weights can be determined by the models’ ability to replicate data. If all models are fitted to the same data using likelihood based methods, weights can be given directly by Akaike or Bayesian Information Criterion (AIC or BIC) [40,41]. In the FMD context, this may be a suitable approach if all included models are data driven kernel models that estimate parameters from outbreak data, such as those proposed by Jewell et al. [42] or Tildesley et al. [43]. However, such weighting schemes would be unfeasible when including detailed simulation models that rely on a large number of parameters, that are determined by expert opinion or lab experiment, such as AusSpread [44], NAADSM [45] and InterSpread Plus [46]. We propose that the future of ensemble modeling for epidemiology will benefit from combining structurally different model types, and methods of weighting need to handle both kernel type models as well as detailed simulation models.

Thus, bias assessment is often confined to the ability of models to replicate observed summary statistics of interest, in particular when the resolution of data observation is on a courser scale than the model prediction [47]. Such methods have been developed within the field of climate forecasting. Giorgi and Mearns [48] introduced a formal framework in which model weights were assessed based on model bias compared to observed data as well as convergence, i.e. agreement with the model consensus. Tebaldi et al. [47] extended the approach to a Bayesian framework. This approach is appealing because it provides probability distributions of quantities of interest, hence uncertainty about the projected outcomes may be provided to policy makers. As such, it would be a suitable approach also for epidemiological predictions.

However, methods developed in one field might not be directly transferable to another. Tebaldi et al. [47] points out that lack of data at fine scale resolution is a limiting factor for climate forecasting. Yet, at courser resolution climate researchers have access to long time series of climate variables to assess model bias. Comparable data may be available for endemic diseases, such as malaria [36] or tuberculosis [49], or seasonally recurrent outbreaks, such as influenza [14] or measles [50]. However, for emerging diseases, long time series would rarely be available, making the lack of data an even bigger issue for epidemiology.

In this methodological paper we aim to explore the potential of using ensemble methods based on the approach presented by Tebaldi et al. [47] for epidemiological projections. The Tebaldi et al. methodology focus on ensembles where projections are made with different models and our aim is to provide a corresponding framework for disease outbreak projections. To investigate the potential of the framework for epidemiology, we here use variations of a single model as a proxy for different models, thus allowing us to investigate how the methodology performs under different levels of discrepancy among projections in the ensemble. We exemplify the implementation by using the UK 2001 FMD outbreak and projections modelled by different parameterizations of the Warwick model [7,9].

In the 2001 UK FMD outbreak, livestock on all infected premises (IPs) were culled. In addition, livestock on farms that were identified to be at high risk of infection were culled as either traditional dangerous contacts (DCs) or contiguous premises (CPs). CPs were defined as “a category of dangerous contact where animals may have been exposed to infection on neighboring infected premises” [5,8]. We start by focusing on ensemble prediction of epidemic duration under the control action employed during the 2001 outbreak compared with an alternative action that excludes CP culling. We investigate sensitivity to priors and explore a hierarchical Bayesian extension of the method to circumvent potential problems with prior sensitivity. We also explore the potential of including subjective a priori trust in the different modeling assumptions and extend the methodology further to allow incorporation of multiple epidemic quantities, here exemplified by adding number of infected and culled farms to the analysis. Through a simulation study, we finally explore the capacity and limitations of the proposed ensemble method, pinpointing some important features of ensemble modeling

## Materials and Methods

**Fig 1:**
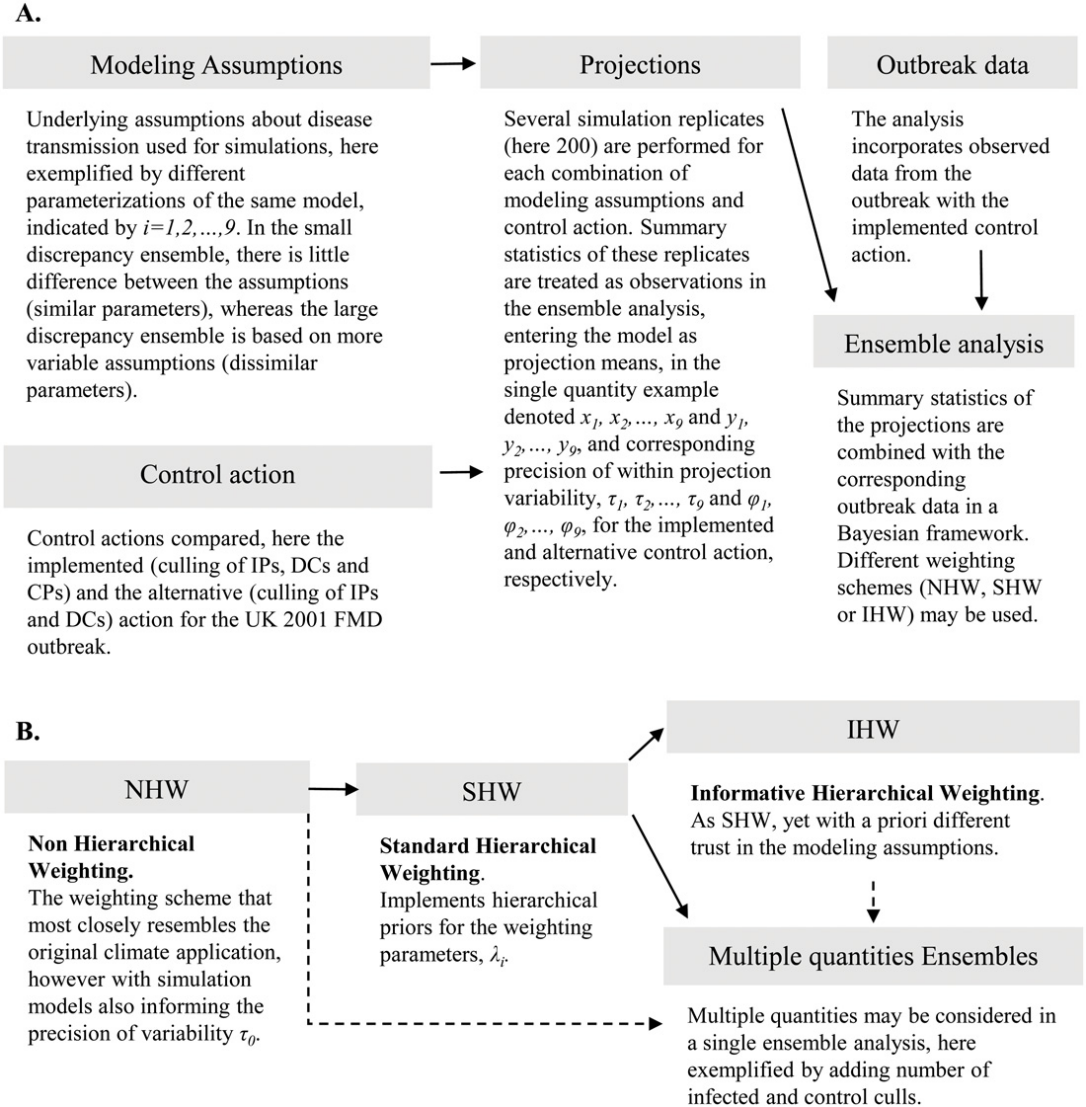
Overview of analyses and methods developments. Panel A presents a conceptual flowchart, showing that modeling assumptions are combined with control actions to simulate projections. These are then combined with observed outbreak data in the ensemble analysis. Panel B presents the methods developments in this study, indicating that we start with the Non Hierarchical Weighting (NHW) scheme, which is most similar to the original climate application. We then extend this to the Standard Hierarchical Weighting (SHW) scheme, and subsequently to Informative Hierarchical Weighting (IHW). We also extend the analysis to consider multiple epidemic quantities. The dashed lines indicate combinations for which the method and the supplied algorithm (S1 File) can be used but are not treated explicitly in the presented examples.

We apply a terminology such that control actions refers to different strategies for disease control. In the ensemble, each action is simulated under different modeling assumptions about the underlying process, expressed as either different models or, as in the example described here, different parameterizations of the same model. We refer to the combination of control action and modeling assumption as a projection. Each projection is further simulated with several replicates, which produce different outcomes merely due to the stochasticity of simulation models. We are also interested in how discrepancy among projections influences the performance of the weighting method and refer to sets of different projections as different ensembles with small and large discrepancy. A flow chart that demonstrates the relationship between different concepts and weighting schemes are presented in Fig 1.

### The Warwick model, control actions and ensembles

We focus on projections of FMD made by the Warwick model [7,9]. This model was developed in the early stages of the 2001 FMD outbreak by Keeling and coworkers to determine the potential for disease spread and the impact of intervention strategies [9]. Here, we utilized a modified version of the model used in 2001, and we briefly describe relevant aspects of the Warwick model with regard to ensemble modeling. Full details of the model can be found in [7,9]. The rate at which an infectious farm *I* infects a susceptible farm *J* is given by:

$$Rate_{IJ}=Sus_{J}\times Trans_{I}\times K(d_{IJ})$$ (1)

where

$$Sus_{J}=({\left[Z_{sheep,J}\right]}^{p_{s,S}}S_{sheep}+{\left[Z_{cow,J}\right]}^{p_{c,S}}S_{cow})$$ (2)

is the susceptibility of farm *J* and

$$Trans_{I}=({\left[Z_{sheep,I}\right]}^{p_{s,T}}T_{sheep}+{\left[Z_{cow,I}\right]}^{p_{c,T}}T_{cow})$$ (3)

is the transmissibility of farm *I* and *K*(*d*_*IJ*_) is the distance-dependent transmission kernel, estimated from contact tracing [9]. In this model *Z*_*s*, *I*_ is the number of livestock species *s* (sheep or cow) recorded as being on farm *I*, *S*_*s*_ and *T*_*s*_ measure the region and species-specific susceptibility and transmissibility, *d*_*IJ*_ is the distance between farms *I* and *J* and *K*(*d*_*IJ*_) is the distance dependent transmission kernel. The parameters, *p*_*s*, *S*_, *p*_*c*, *S*_, *p*_*s*, *T*_ and *p*_*c*, *T*,_ are power law parameters that account for a non-linear increase in susceptibility and transmissibility as animal numbers on a farm increase. Previous work has indicated that a model with power laws provides a closer fit to the 2001 data than when these powers are set to unity [43,51,52].

This version of the model has previously been parameterized to fit to the 2001 FMD outbreak [43]. Region-specific transmissibility and susceptibility parameters (and associated power laws) capture specific epidemiological characteristics and policy measures used in the main hot spots of Cumbria, Devon and the Welsh and Scottish borders. The model is therefore fitted to five regions: Cumbria, Devon, Wales, Scotland and the rest of England (excluding Cumbria and Devon). A table listing all the parameter values used in this model is given in Tildesley et al. [43].

In order to obtain multiple modeling assumptions for ensemble modeling, we specified different transmission rates, *i* as

$$Rate_{IJi}=Sus_{J}\times k_{1i}Trans_{I}\times K(k_{2i}d_{IJ})$$ (4)

where *k*_*1i*_ and *k*_*2i*_ are constants, specific for each modeling assumption, that scale the transmissibility and the spatial kernel, respectively. *k*_*1i*_ = *k*_*2i*_ = 1, follow the parameterizations of Tildesley et al. [43] and by decreasing or increasing these constants, we obtain parameterizations that correspond to best or worst case expectations about the transmissibility and spatial range of transmission. We are interested in how the level of discrepancy among modeling assumptions influences the performance of the ensemble method and we therefore created two different ensembles with different *k*_*1i*_ and *k*_*2i*_, as listed in Table 1. We refer to these as the large and small discrepancy ensemble, corresponding to large and small differences, respectively, in the underlying modeling assumptions used for projections.

**Table 1:** Scaling constants *k1*_*i*_ and *k2*_*i*_ used for each modeling assumption *i* in the small and large discrepancy ensemble.

Modeling assumption (*i*)	Small discrepancy		Large discrepancy	
	*k*_1*i*_	*k*_2*i*_	*k*_1*i*_	*k*_2*i*_
1	1	1	1	1
2	0.95	1	0.8	1
3	1.05	1	1.2	1
4	1	0.95	1	0.8
5	0.95	0.95	0.8	0.8
6	1.05	0.95	1.2	0.8
7	1	1.05	1	1.2
8	0.95	1.05	0.8	1.2
9	1.05	1.05	1.2	1.2

DCs in our model were determined based upon both prior infection by an IP and future risk of infection in the same way as in previous work [8]. CPs were defined as farms that share a common boundary and were determined on an individual basis. The model was seeded with the farms that were predicted to be infected prior to the introduction of movement restrictions on the 23^rd^ February. For each modeling assumption *i* and control action, 200 replicates were simulated and each simulation progressed until the epidemic died out.

### Adapting the Tebaldi et al. method for emerging diseases

To demonstrate concepts and explore the potential of using the Tebaldi et al. [47] approach for epidemiological considerations we initially focus on outbreak duration. This is often considered to be the most costly aspect of FMD outbreaks due to its implication for trade [53]. In section 2.7 we extend the methodology to multiple epidemic quantities. However, the outbreak duration example offers transparent transition from the original climate analysis of Tebaldi et al. [47] that considers the ensemble estimated difference between current and future mean temperatures. In order to introduce the framework to epidemiology, we consider the difference between the implemented and an alternative control action, attempting to show whether the inclusion of CP culling was an appropriate choice given the conditions at the start of the outbreak. As this is a post outbreak analysis, we know the final outbreak duration of the observed outbreak, but that is just a single realization and due to the stochastic nature of disease transmission, the exact outcome may be quite variable. We also have no observed outbreak under the alternative control action to compare with the implemented control. Under these conditions, the most appropriate quantities to compare are the mean duration of a large number of outbreaks under the two control actions, something that can only be achieved through epidemic modeling.

We are interested in comparing projections under the implemented control action to the observed data in order to estimate model weights. Using the Bayesian method of Tebaldi et al. [47], weights as well as statistics of the outbreak, like duration, are considered unknown random variables, and we denote the mean outbreak duration under the implemented and the alternative control action as *μ* and *v*, respectively, corresponding to the mean current and future temperature, respectively, for the climate application. In order to fit with the normal assumptions of the method, we consider log-duration in the analysis.

Weights are expressed through precision, ***λ*** = *λ*_*1*_, *λ*_*2*_,…, *λ*_*n*_, with *λ*_*i*_ denoting the precision of modeling assumption *i*. The projection specific parameter *x*_*i*_ indicates the mean of all replicates under the implemented control action (analog of current climate) for modeling assumption *i*. For the UK 2001 outbreak this included culling of IPs, DCs and CPs. The corresponding projection for the alternative control action (analog of future climate), that included culling of IPs and DCs is denoted *y*_*i*_. The relationship between projections and ensemble parameters is expressed as

$$x_{i}\sim \text{Normal}(\mu ,\lambda_{i}^{-1})$$ (5)

$$y_{i}\sim \text{Normal}(\nu +\beta (x_{i}-\mu ),{\left(\theta \lambda_{i}\right)}^{-1}),$$ (6)

with Normal(*μ*,λ_*i*_^-1^) denoting the normal distribution with mean *μ* and variance λ_*i*_^-1^. Parameter *θ* is included to allow for difference in overall precision of the modeling assumptions under implemented and alternative control actions. However, since projections *x*_*i*_ and *y*_*i*_ are based on simulations, it is fair to assume that modeling assumption *i* that has a high precision for the observed control action also will do well for the unobserved action. This is incorporated by the λ_*i*_ term in both Eqs 5 and 6. For the same reason, we may expect that a projection of a large *x*_*i*_ also corresponds to a large value for *y*_*i*_ and thus *β* is included to allow for correlation between corresponding projections for the two control actions; a projection that e.g. over-predicts duration of the outbreak for the observed control action can be expected to also over-predict the alternative control action.

The analysis of Tebaldi et al. [47] also assessed bias of projections by their ability to reproduce observed current climate by incorporating the relationship between observed current climate *x*_*0*_, an unobserved true mean climate variable (*μ*) and the precision of natural variability *τ*_*0*_ through

$$x_{0}\sim \text{Normal}(\mu ,\tau_{0}^{-1}).$$ (7)

In climate modeling, it is a fair assumption that *τ*_*0*_ is a known, fixed parameter because it can be assessed through historical data. That would rarely be the case for the corresponding epidemic considerations, at least for emerging diseases. Using a single outbreak to evaluate bias, we clearly have no way of assessing variability in outcomes. We therefore include *τ*_*0*_ as an unknown, random variable that is estimated in the analysis as described in the following section.

To aid the interpretation and transfer from the climate to the epidemiological interpretation, we have included Table 2 that lists the variables used in the analysis.

**Table 2:** Variables in the NHW analysis with the interpretation for the original climate interpretation and the epidemiological counterpart.

Variable	Climate Interpretation	Epidemiological Interpretation
*x*_*i*_	Current temperature mean for modeling assumption *i*.	Mean log-duration under implemented control for modeling assumption *i*.
*y*_*i*_	Future temperature mean for modeling assumption *i*.	Mean log-duration under alternative control for modeling assumption *i*.
*x*_*0*_	Observed current temperature.	Observed outbreak log-duration.
*τ*_0_	Precision of natural variability.	Precision of variability of individual outbreaks given the observed initial conditions. *
*μ*	Current mean temperature. *	Mean outbreak duration, implemented control. *
*V*	Future mean temperature. *	Mean outbreak duration, alternative control. *
λ_i_	Precision of model *i* (weights). *	Precision of model *i* (weights). *
*Β*	Correlation between current and. future projections. *	Correlation between implemented and alternative control projections. *
*θ*	Ratio between precision for current and future projections. *	Ratio between precision for implemented and alternative control projections. *
*τ*_i_ and *φ*_i_	Not included.	Precision of within projection variability, calculated as the precision of individual replicates around the mean values *x*_*i*_ and *y*_*i*_.

* Asterisk indicates parameters treated as random variables and no asterisk indicate data.

### Stochasticity and variability

Our main interest in terms of outcome under the implemented control action is *μ* rather than *x*_*0*_. However, it is clear that in addition to the mean duration of the outbreak, the uncertainty about the process also results in some variability in the outcomes that we need to consider. The stochastic simulations used to generate projections provide not only a mean simulated outbreak quantity, but also a range of outcomes that projects the variability. In the absence of repeated outbreaks to evaluate variability of outcomes, an obvious choice would be to use this information to inform the variability *τ*_*0*_. Defining the variability *τ*_*i*_ as the precision of projections under the implemented action for modeling assumptions *i* = 1,2,…,*n* we include a hierarchical structure in the analysis so that for *i* = 0,1,2,…,*n*

$$\tau_{i}\sim \text{Gamma}(a_{\tau },b_{\tau }),$$ (8)

where Gamma(*a*_*τ*_, *b*_*τ*_) indicates the gamma distribution with shape parameter *a*_*τ*_ and rate *b*_*τ*_ both of which are unknown parameters and are estimated in the analysis. Thus, as it would be cumbersome to elicit a fixed prior for *τ*_*0*_ based on our prior expectations about variability, we instead assume that *τ*_*0*_ comes from some, unknown distribution, and make use of *τ*_*1*_, *τ*_*2*_,…, *τ*_*n*_ to inform what this distribution should be.

Similarly, we need to model the variability of projections under the alternative control action, and denoting this *φ*_*i*_ we specify

$$\varphi_{i}\sim \text{Gamma}(a_{\varphi },b_{\varphi }),i=1,2,\ldots ,n$$ (9)

The parameters *a*_*φ*_ and *b*_*φ*_ are conditionally independent from all other parameters in the analysis and can be modelled separately in the Bayesian analysis. As *x*_*i*,_ *y*_*i*_, *τ*_*i*_ and *φ*_*i*_ are calculated from a finite number of realization with each modeling assumption and control action, there is some uncertainty related to this. Tebaldi et al. [47] however points out that while it is certainly possible to construct a Bayesian model that takes this uncertainty into account, the effect is minimal if the number of replicates is large. With the *R* = 200 replicates preformed here, the uncertainty of the mean will in practice have very little effect, and we have included *x*_*i*_, *y*_*i*_, *τ*_*i*_ and *φ*_*i*_ as fixed observations.

Priors for *a*_*τ*_ and *b*_*τ*_ were specified as a gamma distribution with shapes *A*_*aτ*_ and *A*_*bτ*_, respectively, and rates *B*_*aτ*_ and *B*_*bτ*_, respectively. Similarly, the priors for *a*_*φ*_ and *b*_*φ*_, were specified as a gamma distribution with shapes *A*_*aφ*_ and *A*_*bφ*_, respectively, and rates *B*_*aφ*_ and *B*_*bφ*_, respectively. We explored different parameter choices for the hyperpriors and found that the results were insensitive to the choice of prior for a wide range of values. In the analysis presented, we used *A*_*aτ*_ = *A*_*bτ*_ = *B*_*aτ*_ = *B*_*bτ*_ = *A*_*aϕ*_ *A*_*bϕ*_ = *B*_*aϕ*_ = *B*_*bϕ*_ = 0.001. This corresponds to prior distributions with a mean of one and a variance of 1000, thus allowing for a wide range of plausible values.

### Bayesian model

Bayesian analysis requires the specification of prior parameters. We follow Tebaldi et al. [47] with priors specified as uniform on the real line for *μ*, *ν*, and *β*, and λ_*i*_~Gamma(*a*_λ_, *b*_λ_) for *i =* 1,2,…, *n* and *θ*~Gamma(*a*_*θ*_, *b*_*θ*_). We also need to specify hyperpriors for *a*_*τ*_ and *b*_*τ*_, and we implement *a*_*τ*_~Gamma(*A*_*aτ*_, *B*_*aτ*_) and *b*_*τ*_~Gamma(*A*_*bτ*_, *B*_*bτ*_). Denoting ***x* =** *x*_*1*_, *x*_*2*_,…, *x*_*n*_ and ***y* =** *y*_*1*_, *y*_*2*_,…, *y*_*n*_, the full posterior distribution under these priors is given by

$$\begin{array}{l} P\left(\mu ,\nu ,\beta ,\lambda ,\theta ,\tau_{0}|x_{0},x,y,\tau_{1},\tau_{2},\ldots \right)\\ \propto \overset{n}{\underset{i=1}{\prod }}\left(\lambda_{i}^{a_{\lambda }-1}e^{-b_{\lambda }\lambda_{i}}\lambda_{i}\theta^{1/2}\text{exp}\left\{-\frac{\lambda_{i}}{2}\left[{\left(x_{i}-\mu \right)}^{2}+\theta {\left(y_{i}-\nu -\beta \left(x_{i}-\mu \right)\right)}^{2}\right]\right\}\right)\\ \theta^{a_{\theta }-1}e^{-b_{\theta }\theta }\tau_{0}^{1/2}\text{exp}\left\{\frac{\tau_{0}}{2}{\left(x_{0}-\mu \right)}^{2}\right\}\overset{n}{\underset{i=0}{\prod }}\left(\tau_{i}^{a_{\tau }-1}e^{-b_{\tau }\tau_{i}}\right)a_{\tau }^{A_{a\tau }-1}e^{-B_{a\tau }a_{\tau }}b_{\tau }^{A_{b\tau }-1}e^{-B_{b\tau }b_{\tau }} \end{array}$$ (10)

This posterior only differs from the one defined by Tebaldi et al. in that we include *τ*_*0*_ as an unknown variable and use a hierarchical structure for its prior. Using Markov Chain Monte Carlo (MCMC) techniques as described in 2.9, we first performed the analysis with priors as specified by Tebaldi et al. [47] where applicable, i.e. *a*_*λ*_ = *b*_*λ*_ = *a*_*θ*_ = *b*_*θ*_ = 0.001, because they argue that this ensures that the prior contributes little to the posteriors.

However, we propose that this argument is not necessarily always valid. In particular *λ*_*i*_ could be expected to be sensitive to priors because it is essentially only fitted to two data points, *x*_*i*_ and *y*_*i*_. Yet, based on approximations of conditional distributions, Tebaldi et al. argued that the gamma distribution with *a*_*λ*_ = *b*_*λ*_ = 0.001 is appropriately vague for the analysis. For transparency we here follow their approach and investigate the effect of the prior for the simplified model where *β* = 0. The mean of the conditional distribution of *λ*_*i*_ is then approximated by

$$E\left(\lambda_{i}|X_{0},X,Y\right)≅\frac{a_{\lambda }+1}{b_{\lambda }+\frac{1}{2}\left[{\left(x_{i}-\tilde{\mu }\right)}^{2}+\theta {\left(y_{i}-\tilde{\nu }\right)}^{2}\right]},$$ (11)

where $\tilde{\mu }$ is the conditional mean of the distribution of *μ*, given by

$$\tilde{\mu }=(\sum_{i=1}^{n}\lambda_{i}X_{i}+\tau_{0}x_{0})/(\sum_{i=1}^{n}\lambda_{i}+\tau_{0})$$ (12)

and $\tilde{\nu }$ the corresponding value for *v*, given by

$$\tilde{\nu }=(\sum_{i=1}^{n}\lambda_{i}y_{i})/(\sum_{i=1}^{n}\lambda_{i}).$$ (13)

We stress that Σ*λ*_*i*_ need not sum to one, as might be intuitive when using weights. As given by Eqs 11 and 12, the mean of *μ* and *ν* only depends on the relative values of *λ*_*i*_, but the absolute values influence the width of the distribution, with the variance of the conditional distributions increasing with lower absolute values of *λ*_*i*_ (Table 3).

**Table 3:** Conditional distributions used for updating via Gibbs sampling for the single quantity example.

Parameter	Conditional distribution
*λ*_*i*_	$\text{Gamma}(a_{\lambda }+1,{\hat{b}}_{\lambda i}+\frac{{\left(x_{i}-\mu \right)}^{2}-\theta {\left(y_{i}-\nu -\beta (x_{i}-\mu )\right)}^{2}}{2})$ for ${\hat{b}}_{\lambda i}=b_{\lambda }$ for all modeling assumptions *i* in the NHW method,${\hat{b}}_{\lambda i}=a_{\lambda }/m_{\lambda }$ for all modeling assumptions *i* in the SHW method and ${\hat{b}}_{\lambda i}=a_{\lambda }/{\hat{m}}_{\lambda i}$ in the IHW method.
*μ*	$\text{Normal}(\overset{-}{\mu },\sigma_{\mu }^{2})$ with $\sigma_{\mu }^{2}={\left(\sum^{}\lambda_{i}+\theta \beta^{2}\sum^{}\lambda_{i}+\tau_{0}\right)}^{-1}$ $\overset{-}{\mu }=\sigma_{\mu }^{2}(\sum^{}\lambda_{i}x_{i}-\theta \beta \sum^{}\lambda_{i}(y_{i}-\nu -\beta x_{i})+\tau_{0}x_{0})$
*v*	$\text{Normal}(\overset{-}{\nu },{\left(\theta \sum^{}\lambda_{i}\right)}^{-1})$ with $\overset{-}{\nu }=\frac{\sum^{}\lambda_{i}(y_{i}-\beta (x_{i}-\mu ))}{\sum^{}\lambda_{i}}$
*β*	$\text{Normal}(\overset{-}{\beta },\sigma_{\beta }^{2})$ with $\sigma_{\beta }^{2}={\left(\theta \sum^{}\lambda_{i}{\left(x_{i}-\mu \right)}^{2}\right)}^{-1}$ $\overset{-}{\beta }=\sigma_{\beta }^{2}\theta^{-1}\sum^{}\lambda_{i}(y_{i}-\nu )(x_{i}-\mu )$
*θ*	$\text{Gamma}(a_{\theta }+\frac{N}{2},b_{\theta }+\frac{\sum^{}\lambda_{i}{\left(y_{i}-\nu -\beta (x_{i}-\mu )\right)}^{2}}{2})$
*τ*_*0*_	$\text{Gamma}(a_{\tau }+1/2,b_{\tau }+\frac{{\left(x_{i}-\mu \right)}^{2}}{2})$
*φ*_*0*_	Gamma(*a*_*ϕ*_, *b*_*ϕ*_)

While a low value of *a*_*λ*_ certainly ensures little contribution to the numerator in Eq (11), it is less evident that a low value for *b*_*λ*_ contributes little to the denominator because if $x_{i}\to \tilde{\mu }$ and $y\to \tilde{\nu }$, the denominator actually approaches *b*_*λ*_. Hence, to ensure that a low value of *b*_*λ*_ can be considered vague such that our posterior is informed primarily by *x*_0_, ***x*** and ***y***, we must conclude that $\left|x_{i}-\tilde{\mu }\right|$ or $\left|y_{i}-\tilde{\nu }\right|$ is clearly separated from zero. However, if *λ*_*i*_≫*λ*_*j*_ for all *i* ≠ *j* and *λ*_*i*_≫*τ*_*0*_, then $\tilde{\mu }\approx x_{i}$ and $\tilde{\nu }\approx y_{i}$ and nothing in the model prevents this relationship. In fact, if we consider the gamma prior with shape and scale parameters set to 0.001, the distribution has most of its density near zero, however with a fat tail (yet exponentially bounded) that allows for high values. In the current analysis, this corresponds to the prior belief that the majority of modeling assumptions will have very low precision while a few will have very high. Under this prior belief, it is expected that for some model *i*, *λ*_*i*_≫*λ*_*j*_ for all *i* ≠ *j*. In the instance where instead *τ*_0_≫*λ*_*i*_ for all *i*, then $\tilde{\mu }\approx x_{0}$ and the approximation would hold, but we cannot expect that relationship.

As we cannot a priori be sure that the choice of *a*_*λ*_ and *b*_*λ*_ does not influence our posterior as long as they are arbitrarily small, we performed a prior sensitivity analysis and re-ran the analysis with *a*_*λ*_ *= b*_*λ*_ *=* 0.01 and *a*_*λ*_ *= b*_*λ*_ *=* 0.0001. We could expect that the sensitivity to priors depends on the difference among modeling assumptions, and we investigate this by analyzing ensembles with little and large discrepancy between assumptions in the ensemble as given by Table 1.

We refer to this as the Non Hierarchical Weighting (NHW) method.

### Standard Hierarchical Weighting model

If we cannot ensure that the analysis is insensitive to the choice of prior, it implies that our prior beliefs will influence how much different projections contribute to ensemble predictions with the current method. Using prior beliefs is sometimes desirable, and in section 2.6 we consider the situation where we trust some modeling assumptions more than others. However, it would rarely be the case that we would have substantial expectations that could inform the shape, *a*_*λ*_, and scale, *b*_*λ*_, of the prior for ***λ***.

A potential solution might be to extend the model to include hierarchical priors such that the prior for *λ*_*i*_ is estimated in the model rather than a priori fixed. We make a slight change to the parameterization of the prior distribution such that

$$\lambda_{i}\sim \text{Gamma}(a_{\lambda },a_{\lambda }/m_{\lambda }),$$ (14)

i.e. specifying the distribution by its mean *m*_*λ*_ and shape *a*_*λ*_, which are estimated in the model. In that way, we move our uncertainty up a level and express our beliefs about the distribution of *m*_*λ*_ and *a*_*λ*_, rather than ***λ***. Using *m*_*λ*_ rather than *b*_*λ*_ facilitates the specification of a prior for the mean precision parameter that corresponds to the priors previously specified on individual *λ*_*i*_. This parameterization further aids the use of prior beliefs about weights in section 2.6.

While we can never be strictly uninformative in Bayesian analysis, the hierarchical prior can allow for a wide range of plausible *m*_*λ*_ and *a*_*λ*_ whereas the model presented in section 2.4 requires these to be specified explicitly. This also allows for the concept of “borrowing strength” [54], such that the distribution of each *λ*_*i*_ can be indirectly informed by all other precisions via the hierarchical distribution. This is often beneficial in situations where individual parameters are fitted to a small amount of data [55,56], which clearly is the case for *λ*_*i*_ here. To extend Eq (10) to a hierarchical model, we include hyperpriors such that

$$a_{\lambda }\sim \text{Gamma}(A_{a\lambda },B_{a\lambda })$$ (15)

and

$$m_{\lambda }\sim \text{Gamma}(A_{m\lambda },B_{m\lambda }).$$ (16)

We performed the corresponding sensitivity analysis for the hierarchical ensemble prediction by applying hyperpriors *A*_*aλ =*_ *B*_*aλ =*_ *A*_*mλ =*_ *B*_*mλ*_ set to 0.01, 0.001 and 0.0001. We refer to this as hierarchical sensitivity set-up one. Secondly, we performed a sensitivity analysis, hierarchical sensitivity set-up two, where we fixed the shape parameters *A*_*aλ =*_ *A*_*mλ =*_ 0.001 and only varied B_*aλ*_ = B_*mλ*_, again set to either 0.01, 0.001 or 0.0001.

We refer to this as model as the Standard Hierarchical Weighting (SHW) method.

### Informative Hierarchical Weighting model

Using expert opinions may substantially improve predictions [57], and there are several instances where incorporating prior beliefs that reflect the “trust” in different modeling assumptions could be useful. For instance, a policymaker might have more trust in one model type over another, and rather than excluding the models that are considered less reliable (i.e. giving them a priori zero weigh), it could be useful to include them, yet with less contribution to the ensemble.

In the case considered here, where modeling assumptions represent most likely, best and worst case in terms of parameterization, we might want to give the “most likely” modeling assumption higher weight. For the analysis with fixed *a*_*λ*_ and *b*_*λ*_, described in section 2.4, we could merely elicit a different scale parameter *b*_*λ*_ for each *λ*_*i*_, such that modeling assumptions with high trust are given a low value. However, with the shape parameter *a*_*λ*_ set to a low value (“vague” shape), the prior may have little effect on the posterior *λ*_*i*_. Eliciting a high value of *a*_*λ*_ would instead result in a posterior that is merely the results of our prior beliefs and we have no foundation for which to elicit some intermediate value.

In order to combine the hierarchical approach with informative priors, we propose a modification of the analysis presented in section 2.5, where the assumption of exchangeability is relaxed in the hierarchical structure with

$$\lambda_{i}\sim \text{Gamma}(a_{\lambda },a_{\lambda }/{\hat{m}}_{\lambda i}),$$ (17)

where ${\hat{m}}_{\lambda i}=w_{i}m_{\lambda }$ and *w*_*i*_ indicates the a priori trust in modeling assumption *i*. With *w*_*i*_ = *kw*_*j*_, the prior distribution of *λ*_*i*_ will have a mean that is *k* times that of *λ*_*j*_ and from Eqs (12) and (13) the relationship also implies that before ***λ*** is estimated (i.e. involving the data *x*_*0*_, ***x*** and ***y***), the outputs of modeling assumption *i* will contribute *k* times as much to *μ* and *v* as does assumption *j*.

To demonstrate the effect that a priori trust in different modeling assumptions can have on the posterior estimates, we consider the case where the best, most likely and worst case scenarios for each of the two varied parameters corresponds to percentile 2.5, 50 and 97.5, respectively, of a normal distribution. Given that the density at percentiles 2.5 and 97.5 then is 0.15 of that at the mode, we specify *w*_*i*_ = 0.15 for *i* = 2, 3, 4 and 7, i.e. for modeling assumptions where one of the varied parameters follows the most likely scenario, whereas the other one is set to either worst or best case. With the same rationality, we specify *w*_*i*_ = 0.021 for *i* = 5, 6, 8 and 9, i.e. modeling assumptions where both parameters follow either best or worst case expectations.

We also investigate the case where a high weight is given to a projection *x*_*i*_ further away from the observed data *x*_*0*_. Consistently, modeling assumptions *i* = 5 predicted the shortest duration for all actions and ensembles. We therefore also performed the analysis with *w*_*5*_ = 1 and *w*_*1*_ = 0.021, and all other weights are as above. This allows us to investigate the performance of the informative weighting method when an outlier is up-weighted.

We refer to this method as the Informative Hierarchical Weighting (IHW) method.

### Multiple epidemic quantities

In the above examples, we focused on a single epidemic quantity, allowing for transparent transition from the original Tebaldi et al. work [47] that focused on temperature. For epidemiology, it may however be useful to consider multiple epidemic quantities. This could be done in different ways, but here we offer a straightforward multi-quantity extension of the Bayesian model for the single epidemic quantity, based on the supposition that the relative weights are equal for all quantities. As such, we implement a single weighting parameter *λ*, shared among all quantities. For other parameters, we use a similar notation as for the single quantity analysis, but give many of the parameters an additional index *q*, indicating that the parameter is quantity specific. We expand the Bayesian model by defining

$$x_{i,q}\sim \text{Normal}(\mu_{q},\theta_{\mu ,q}\lambda_{i}^{-1}),$$ (18)

$$y_{i,q}\sim \text{Normal}(\nu_{q}+\beta_{q}(x_{i,q}-\mu_{q}),{\left(\theta_{\nu ,q}\lambda_{i}\right)}^{-1}),$$ (19)

$$x_{0,q}\sim \text{Normal}(\mu_{q},\tau_{0,q}^{-1}),$$ (20)

where *x*_*i*, *q*_ and *y*_*i*, *q*_ are the mean projections of modeling assumption *i* for epidemic quantity *q* for the implemented and alternative control action, respectively, and *x*_*0*, *q*_ is the corresponding observed value. As for the single epidemic quantity example, *μ*_*q*_and *V*_*q*_are the expected values of quantity *q*, and because we cannot expect to have the same correlation between control actions for all quantities, *β*_*q*_ is included as unique for each *q*. Parameters *θ*_*μ*, *q*_ and *θ*_*v*, *q*_ scales the precision of models between actions and quantities and the parameters of the Bayesian model are identifiable by defining *θ*_*μ*, *1*_ = 1. Similarly, we specify quantity specific parameters

τi,q~Gamma(aτ,q,bτ,q),i=0,1,2,…,n,φi,q~Gamma(aφ,q,bφ,q),i=1,2,…,n. (21)

The conditional distributions for the multi-quantity extension are provided in Table 4. We denote the total number of quantities in an analysis as *Q*.

**Table 4:** Conditional distributions used for updating via Gibbs sampling for *Q* epidemic quantities.

Parameter	Conditional distribution
*λ*_*i*_	$\text{Gamma}(a_{\lambda }+Q,{\hat{b}}_{\lambda i}+\sum_{q=1}^{Q}\frac{\theta_{\mu ,q}{\left(x_{i,q}-\mu_{q}\right)}^{2}-\theta_{\nu ,q}{\left(y_{i,q}-\nu_{q}-\beta_{q}(x_{i,q}-\mu_{q})\right)}^{2}}{2})$ for ${\hat{b}}_{\lambda i}=b_{\lambda }$ for all modeling assumptions *i* in the NHW method,${\hat{b}}_{\lambda i}=a_{\lambda }/m_{\lambda }$ for all modeling assumptions *i* in the SHW method and ${\hat{b}}_{\lambda i}=a_{\lambda }/{\hat{m}}_{\lambda i}$ in the IHW method.
*μ*_*q*_	$\text{Normal}(\overset{-}{\mu },\sigma_{\mu }^{2})$ with $\sigma_{\mu }^{2}={\left(\sum^{}\theta_{\mu ,q}\lambda_{i}+\theta_{\nu ,q}\beta_{q}^{2}\sum^{}\lambda_{i}+\tau_{0,q}\right)}^{-1}\overset{-}{\mu }=\sigma_{\mu }^{2}(\sum^{}\theta_{\mu ,q}\lambda_{i}x_{i,q}-\theta_{\nu ,q}\beta_{q}\sum^{}\lambda_{i}(y_{i,q}-\nu_{q}-\beta_{q}x_{i,q})+\tau_{0,q}x_{0,q})$
*v*_*q*_	$\text{Normal}(\overset{-}{\nu },{\left(\theta_{\nu ,q}\sum^{}\lambda_{i}\right)}^{-1})$ with $\overset{-}{\nu }=\frac{\sum^{}\lambda_{i}(y_{i,q}-\beta_{q}(x_{i,q}-\mu_{q}))}{\sum^{}\lambda_{i}}$
*β*_*q*_	$\text{Normal}(\overset{-}{\beta },\sigma_{\beta }^{2})$ with $\sigma_{\beta }^{2}={\left(\theta_{\nu ,q}\sum^{}\lambda_{i}{\left(x_{i,q}-\mu_{q}\right)}^{2}\right)}^{-1}$ $\overset{-}{\beta }=\sigma_{\beta }^{2}\theta_{\nu ,q}^{-1}\sum^{}\lambda_{i}(y_{i,q}-\nu_{q})(x_{i,q}-\mu_{q})$
*θ*_*μ*, *q*_ for *q* ≠ 1	$\text{Gamma}(a_{\theta }+\frac{N}{2},b_{\theta }+\frac{\sum^{}\lambda_{i}{\left(x_{i,q}-\mu_{q}\right)}^{2}}{2})$
*θ*_*v*, *q*_	$\text{Gamma}(a_{\theta }+\frac{N}{2},b_{\theta }+\frac{\sum^{}\lambda_{i}{\left(y_{i,q}-\nu_{q}-\beta_{q}(x_{i,q}-\mu_{q})\right)}^{2}}{2})$
*τ*_*0*, *q*_	$\text{Gamma}(a_{\tau ,q}+1/2,b_{\tau ,q}+\frac{{\left(x_{i,q}-\mu_{q}\right)}^{2}}{2})$
*φ*_*0*, *q*_	Gamma(*a*_*ϕ*, *q*_, *b*_*ϕ*, *q*_)

### Analysis of simulated outbreaks

The above examples focus on the UK 2001 FMD outbreak and show how the introduced framework can be applied to actual outbreak data. However, a limitation to this approach is that we are confined to investigating the behavior of the ensemble methodology for that particular outbreak. To further investigate the potential and limitations of the proposed methods, we also performed analysis of simulated outbreak data. With simulated data, we have “true” estimates of *μ* and *v*, and we want to explore the ability of the ensemble to predict these under two different conditions; when the true values lies within the range of **X** and **Y** predicted by the individual models of the ensemble and when it does not. For multi-model ensembles, this corresponds to the situation where the true behavior of the outbreak is encapsulated within the range of underlying assumptions of the individual models and when it is not.

Here we explore the outcome of these conditions by first simulating outbreaks with the parameterizations of modeling assumption 1 (*k*_1_ = *k*_2_ = 1), i.e. located in the center of both the small and large discrepancy ensemble. This simulates outbreaks where the true behavior of the outbreak is encapsulated within the range of underlying assumptions of the individual projections for both ensembles. We also simulate outbreaks with a parameterization where both *k*_1_ and *k*_2_ are set to 0.9. This produces outbreaks where the true behavior is outside of the assumptions of the projections for the small discrepancy ensemble, yet inside the range of the large discrepancy ensemble.

The exact behavior of the ensemble depends on the actual realization of the individual outbreak, because the observed values *x*_*0*_ are different due to the stochastic disease transmission process. We therefore apply both the small and large discrepancy ensembles to ten realizations of each of the simulation parameterizations. We implement both the single and multiple epidemic quantity analysis, thus further highlighting the effect of using multiple quantities.

### Computation

We use Markov Chain Monte Carlo (MCMC) techniques to obtain samples from the full posterior distribution of the proposed Bayesian models (NHW, SHW and IHW). For many parameters, the conditional distribution belongs to a standard parametric family, thus allowing for Gibbs sampling. We list these conditional distributions in Table 3 for single quantity analysis and Table 4 for multiple quantities.

We also rely on Metropolis-Hastings (M-H) updates, and with the computation used for multi-quantity analysis being a straightforward extension of that used for the single quantity, we start by describing the update scheme for the single quantity analysis. The conditional distribution of *b*_*τ*_ has a known form, $P(b_{\tau }\text{|}\ldots )=\text{Gamma}(A_{b\tau }+(N+1)a_{\tau },B_{b\tau }+\sum_{i=0}^{N}\tau_{i})$, that would allow for Gibbs sampling of *b*_*τ*_, whereas M-H updates need to be implemented for *a*_*τ*_. We however found strong correlation between the marginal posterior estimates of *a*_*τ*_ and *b*_*τ*_, and mixing was improved by performing joint M-H updates of these parameters by multivariate Random Walk (RW) proposals. Mixing can be further improved by updating parameters on a transform that resembles a Gaussian distribution, and we therefore performed updates on the log-transform, i.e. based on current values of *a*_*τ*_ and *b*_*τ*_ We proposed candidate parameters $\left[log(a_{\tau }^{*}),\text{log}(b_{\tau }^{*})\right]$ from MVN([log(*a*_*τ*_),log(*b*_*τ*_)],Σ_*τ*_). Here MVN indicates the multivariate normal distribution and *Σ*_*τ*_ the covariance matrix. Candidate values are accepted with the probability

$$\text{min}(1,\frac{\text{Gamma}(a_{\tau }^{*}\text{|}A_{a\tau },B_{a\tau })\text{Gamma}(b_{\tau }^{*}\text{|}A_{b\tau },B_{b\tau })\prod_{i=0}^{N}\text{Gamma}(\tau_{i}\text{|}a_{\tau }^{*},b_{\tau }^{*})}{\text{Gamma}(a_{\tau }\text{|}A_{a\tau },B_{a\tau })\text{Gamma}(b_{\tau }\text{|}A_{b\tau },B_{b\tau })\prod_{i=0}^{N}\text{Gamma}(\tau_{i}\text{|}a_{\tau },b_{\tau })}|J_{\tau }|),$$ (22)

where $|J_{\tau }|=a_{\tau }^{*}b_{\tau }^{*}{\left(a_{\tau }b_{\tau }\right)}^{-1}$ indicates the Jacobian determinant of the log-transform.

Mixing can be improved if the covariance matrix *Σ*_*τ*_ is proportional to the covariance of the marginal posterior of [log(*a*_*τ*_),log(*b*_*τ*_)], here indicated as *Φ*. However, this is not known prior to the analysis. We therefore implement an optimized method of the Robbins-Monroe search process as presented by Garthwaite et al. [58]. This estimates the covariance during the MCMC and finds the scaling parameter *ρ* such that Σ_*τ*_ = *ρ*Φ provides a chosen long term acceptance rate, here set to 0.234 based on suggestions by Roberts et al. [59]. The method has been demonstrated to be appropriate also for RW on transformed scales of the parameters [60].

The corresponding updates of *a*_*φ*_ and *b*_*φ*_ were also performed with M-H updates and we proposed candidate parameters $\left[log(a_{\varphi }^{*}),\text{log}(b_{\varphi }^{*})\right]$ from MVN([log(*a*_*ϕ*_),log(*b*_*ϕ*_)],Σ_*ϕ*_). and accepted them with probability

$$\text{min}(1,\frac{\text{Gamma}(a_{\varphi }^{*}\text{|}A_{a\varphi },B_{a\varphi })\text{Gamma}(b_{\varphi }^{*}\text{|}A_{b\varphi },B_{b\varphi })\prod_{i=1}^{N}\text{Gamma}(\varphi_{i}\text{|}a_{\varphi }^{*},b_{\varphi }^{*})}{\text{Gamma}(a_{\varphi }\text{|}A_{a\varphi },B_{a\varphi })\text{Gamma}(b_{\varphi }\text{|}A_{b\varphi },B_{b\varphi })\prod_{i=1}^{N}\text{Gamma}(\varphi_{i}\text{|}a_{\varphi },b_{\varphi })}|J_{\varphi }|).$$ (23)

We used a similar approach for updates of *a*_*λ*_ and *m*_*λ*_ in the hierarchical methods (SHW and IHW) and proposed $\left[log(a_{\lambda }^{*}),\text{log}(m_{\lambda }^{*})\right]$ from MVN([log(*a*_λ_),log(*b*_λ_)],Σ_λ_). Candidate parameters were accepted with probability

$$min(1,\frac{\text{Gamma}(a_{\lambda }^{*}\text{|}A_{a\lambda },B_{a\lambda })\text{Gamma}(m_{\lambda }^{*}\text{|}A_{b\lambda },B_{b\lambda })\prod_{i=1}^{N}\text{Gamma}(\lambda_{i}\text{|}a_{\lambda }^{*},{\hat{b}}_{\lambda i}^{*})}{\text{Gamma}(a_{\lambda }\text{|}A_{a\lambda },B_{a\lambda })\text{Gamma}(m_{\lambda }\text{|}A_{b\lambda },B_{b\lambda })\prod_{i=1}^{N}\text{Gamma}(\lambda_{i}\text{|}a_{\lambda },{\hat{b}}_{\lambda i})}|J_{\lambda }|),$$ (24)

where ${\hat{b}}_{\lambda i}=a_{\lambda }/m_{\lambda }$ for all modeling assumptions *i* in the SHW method and ${\hat{b}}_{\lambda i}=a_{\lambda }/{\hat{m}}_{\lambda i}$ with ${\hat{m}}_{\lambda i}=w_{i}m_{\lambda }$ in the IHW method. As above, we used the method of Garthwaite et al. [58] to determine Σ_λ_ to obtain a long term acceptance rate of 0.234.

We also found strong correlation between *μ* and *ν*. In order to improve mixing, we repeated the updates of these parameters five times for each iteration of the MCMC.

The same update scheme was used for the multi-quantity consideration, yet with a separate Σ_*τ*, *q*_, Σ_*ϕ*, *q*_ and Σ_*λ*, *q*_ adaptively estimated for each quantity *q*.

The algorithm was implemented in MATLAB (The MathWorks, Inc., Natick, Massachusetts, United States) and code is available as supplementary information (S1 File).

## Results

### Single quantity analysis

**Fig 2:**
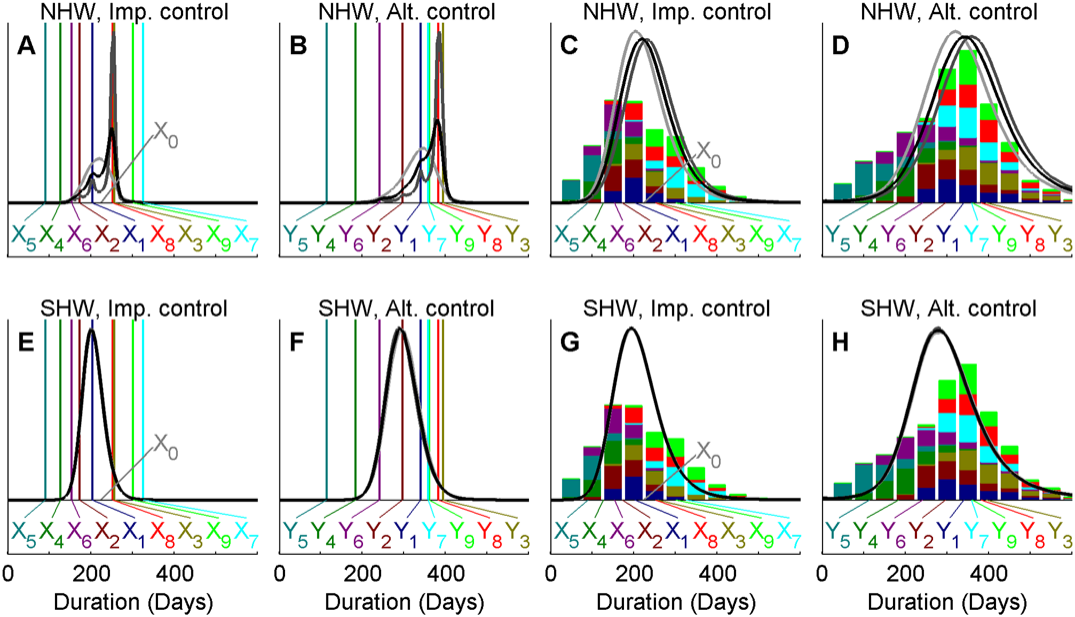
Ensemble prediction for the UK 2001 FMD outbreak duration. Comparing two methods of ensemble prediction of expected outbreak duration under implemented and alternative control actions (panels A, E and B, F, respectively) as well as the corresponding duration of individual outbreaks (panels C, G and D, H, respectively). The observed duration of the UK 2001 FMD outbreak is indicated by *X0* while vertical colored lines (panels A, B, E and F) and annotations *X1*, *X2*,.., *X9* and *Y1*, *Y2*,…, *Y9* indicate the mean outbreak duration of each projection. The total bar height (panels C, D, G, H) indicate the frequency of simulations across all projections with outbreaks ending within each 50 day interval and colors indicate the contribution of each of the projections. Results are shown for analyses of the large discrepancy ensemble using the NHW method (panels A-D) and the SHW method (panels E-H). Marginal posterior estimates correspond to different priors, with prior parameters *aλ = bλ* = 0.01 (light gray), *aλ = bλ* = 0.001 (black) and *aλ = bλ* = 0.0001 (dark gray) the NHW method (panels A-D) and *Aaλ = Abλ = Baλ = Bbλ* set to 0.01 (light gray), 0.001 (black) and 0.0001 (dark gray) for the SHW method (panels E-H). Note that posterior estimates are very similar for the latter, making the lines overlap.

We start by presenting the results for the single quantity analysis, highlighting the behavior of the method for the NHW, SHW and IHW schemes. Fig 2, panels A and B show the estimates of outbreak duration for the two control actions for the large discrepancy ensembles using the NHW method and reveals rather large prior sensitivity. Note that we plot marginal posteriors of *M* = *e* ^*μ*^ and *N* = *e* ^*v*^, respectively. As such, the posteriors represent the geometric mean outbreak duration. The corresponding arithmetic mean can be calculated as *e* ^*μ*+1/(2*τ*^_0_^)^ and *e* ^*v*+1/(2*ϕ*^_0_^)^, respectively, yet we use the geometric mean as it more clearly shows the relationship with individual projections, here presented by *x*_*i*_ = *e* ^*x*^_*i*_ and *Y*_*i*_ = *e* ^*y*^_*i*_, respectively. For *a*_*λ*_ = *b*_*λ*_ = 0.0001 (solid gray lines), the distributions are multimodal with peaks at individual model predictions, whereas a more smooth shape is obtained for *a*_*λ*_ = *b*_*λ*_ = 0.01 (dashed black lines) and *a*_*λ*_ = *b*_*λ*_ = 0.001 (solid black line) yields an intermediate result.

With the SHW method, we instead obtain posteriors that are largely insensitive to the choice of hyperprior. Fig 2, panels E and F present the result of sensitivity set-up one, showing near identical posterior estimates when hyperparameters *A*_*aλ*_, *B*_*aλ*_, *A*_*mλ*_ and *B*_*mλ*_ are set to 0.01, 0.001 or 0.0001. Sensitivity set-up two produced results that were visually indistinguishable from panels E and F and are not presented. This further indicates that the hierarchical method is robust to the choice of hyperpriors.

Within epidemiology, there is clearly an interest in not just the expected outbreak duration, but also other statistics such as the probability of large outbreaks occurring. We therefore consider the posterior predictive distributions of individual outbreak durations under the two control actions. For the non-hierarchical model (Fig 2, panels C and D), there is an obvious effect of the choice of prior with higher probability of long outbreaks for lower values of *a*_*λ*_ and *b*_*λ*_. For the hierarchical model (Fig 2, panels G and H), there is again little difference among posteriors corresponding to different priors.

**Fig 3:**
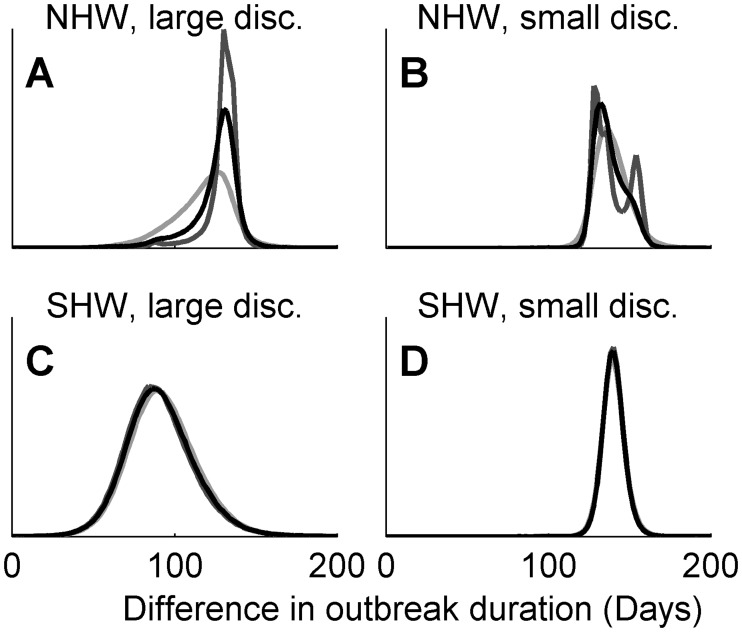
Ensemble predicted difference between control actions. Posterior predictive distributions of the difference in outbreak duration between implemented and alternative control actions for the 2001 UK FMD outbreak using the NHW method (panels A, B) and the SHW method (panels C, D,). Results are shown for large (panels A, C) and small (panels B, D) discrepancy among projections in the ensemble. Marginal posterior estimates correspond to different priors, with prior parameters *aλ = bλ* = 0.01 (light gray), *aλ = bλ* = 0.001 (black) and *aλ = bλ* = 0.0001 (dark gray) for the NHW method (panels A, B) and *Aaλ = Abλ = Baλ = Bbλ* set to 0.01 (light gray), 0.001 (black) and 0.0001 (dark gray) for the SHW method (panels C, D). Due to high similarity of estimates, the plots are largely overlapping for the hierarchical method.

When evaluating the efficiency of control actions, the difference *N-M* is of particular interest. In the example presented here, this estimates how much longer the outbreak would have been if culling of CPs had been excluded from the control. As shown in Fig 3, the estimates are again sensitive to the choice of prior with the NHW method, yet insensitive with the SHW method. The range of the posterior under the NHW method is less sensitive to the prior for the low discrepancy ensemble (panel B) than for the large discrepancy ensemble (panel A), where higher probability of less difference is estimated with *a*_*λ*_ = *b*_*λ*_ = 0.01 than for *a*_*λ*_ = *b*_*λ*_ = 0.0001. However, the multimodal behavior of the NHW method with low values of *a*_*λ*_ and *b*_*λ*_ is obtained also for the low discrepancy ensemble.

**Fig 4:**
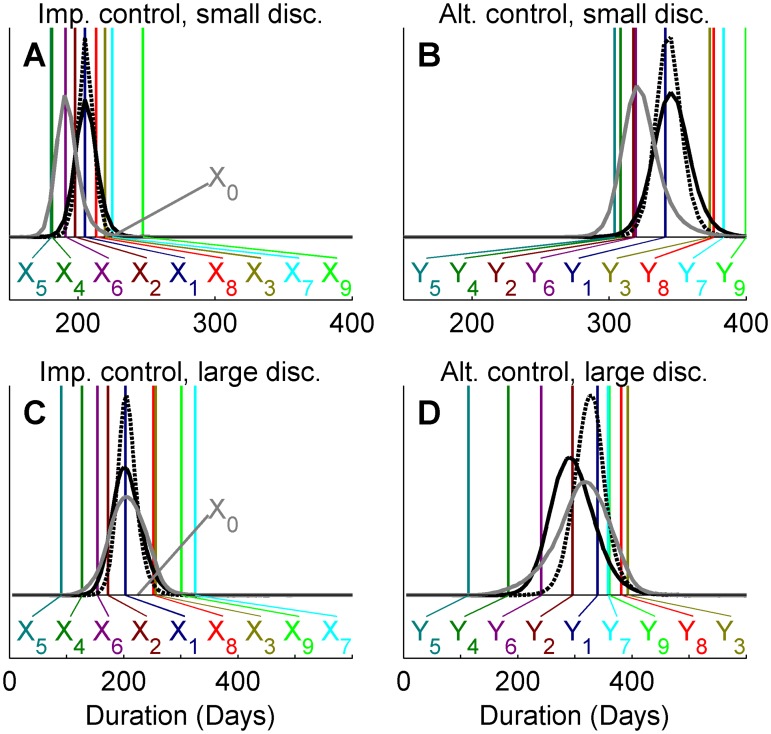
Ensemble prediction of expected outbreak duration with the IHW method. Panels A and C show ensemble estimates of mean duration for the implemented control action of the 2001 UK FMD outbreak under small and large discrepancy ensemble, respectively. Panels B and D shows the corresponding estimates for an alternative control action. Marginal posterior distributions indicate the expected outbreak duration estimated by the ensemble with a priori equal weights (solid black line), up-weighting of the most likely scenarios (*i =* 1; dashed black line), up-weighting of the outlier (*i =* 5; solid gray line). Observed outbreak duration is indicated by *X0* and predicted means under the implemented and alternative control action are indicated by *X1*, *X2*,… *X9* and *Y1*, *Y2*,…, *Y9*, respectively.

Fig 4 demonstrates the effect that a priori beliefs about the weights have on the predicted outbreak duration under large and small discrepancy ensembles. When using a priori higher weights for the most likely scenarios (modeling assumption one; black dotted lines), the posterior estimates are shifted and become more centered on projections of that particular modeling assumption compared to the case where a priori weights are equal (black solid line). The outcome of up-weighting the outlier (modeling assumption five; solid gray lines) is however different between the two ensembles. For the small discrepancy ensemble (panels A and B), similar results are found as for the up-weighting of the most likely scenarios; posteriors are shifted towards the projection with a priori high weight. For the high discrepancy ensemble (panels C and D), the posterior estimates of outbreak duration instead become wider for both control actions, indicating larger uncertainty about the expected duration of outbreaks.

**Fig 5:**
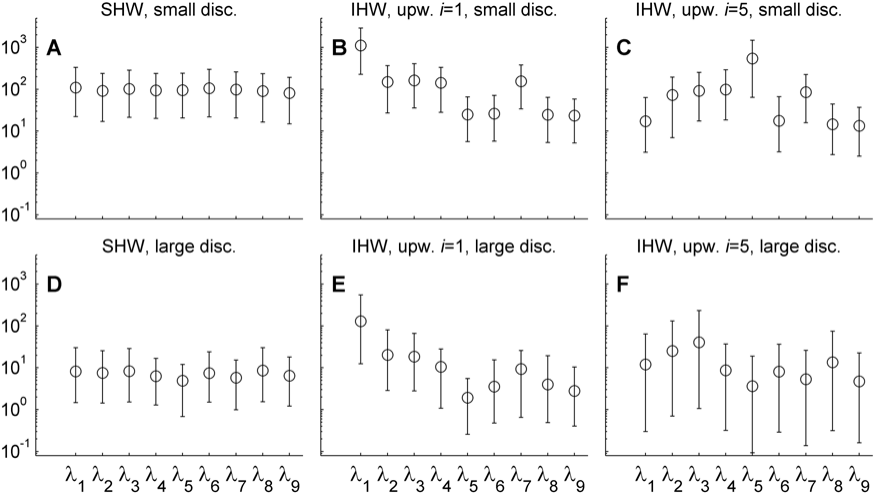
Marginal posterior estimates of weighting parameters, *λ*. Posterior means are indicated with circles and error bars indicate 95% central credibility intervals under a priori equal weights (left column panels), up-weighting of modeling assumption *i* = 1(middle column panels) and up-weighting of assumption *i =* 5 (right column panels). Results are shown for small and large discrepancy ensembles in top and bottom row panels, respectively.

Fig 5 shows the marginal posterior estimates of individual weights *λ*_*i*_ under different discrepancy among projections and informative weighting schemes. When using a priori equal weights, there is little difference in the estimates for the small discrepancy ensemble (top left panel) whereas moderate differences are obtained for large discrepancy (bottom left panel). Note that while the error bars are overlapping, the mean estimate of the most likely scenarios (modeling assumption one) is approximately 1.7 times as large as that of the outlier(modeling assumption five), meaning that the former will contribute approximately 1.7 times as much to the posterior means of *μ* and *v* than the latter (Eqs (12) and (13)).

When giving a priori highest weight to the most likely scenario (modeling assumption one; middle column panels), the posterior estimate of *λ*_*1*_ is consistently shifted upwards, meaning that the most likely scenarios (modeling assumption one) will contribute more to the posteriors of *μ* and *ν* than other projections. For the up-weighting of the outlier, projections corresponding to modeling assumption five, the same is found when there is little discrepancy among projections (lower right panel). This is however not found for the high discrepancy ensemble (top right panel), where the main effect is that compared to equal a priori weights (top left panel), the error bars are wider; this indicates larger uncertainty about weights and consequently about the contribution of individual projections to the posterior estimates of outbreak durations.

### Multiple quantity analysis and simulated outbreaks

**Fig 6:**
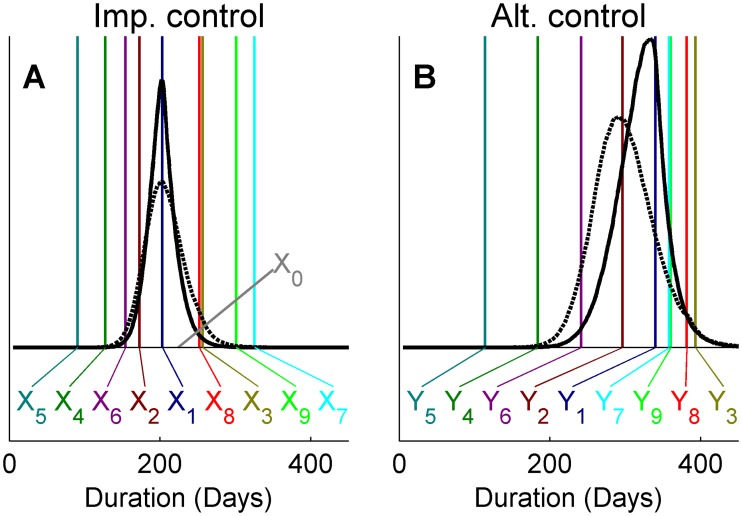
Ensemble prediction of outbreak duration with *Q* = 1 and *Q* = 3. Posterior estimates of mean outbreak duration for the implemented (Panel A) and alternative (Panel B) control action, with the dashed line indicating the posteriors corresponding to the single quantity analysis and the solid line indicating the corresponding posterior when number of infected and control culls are included. The figure shows the result of the large discrepancy ensemble.

**Fig 7:**
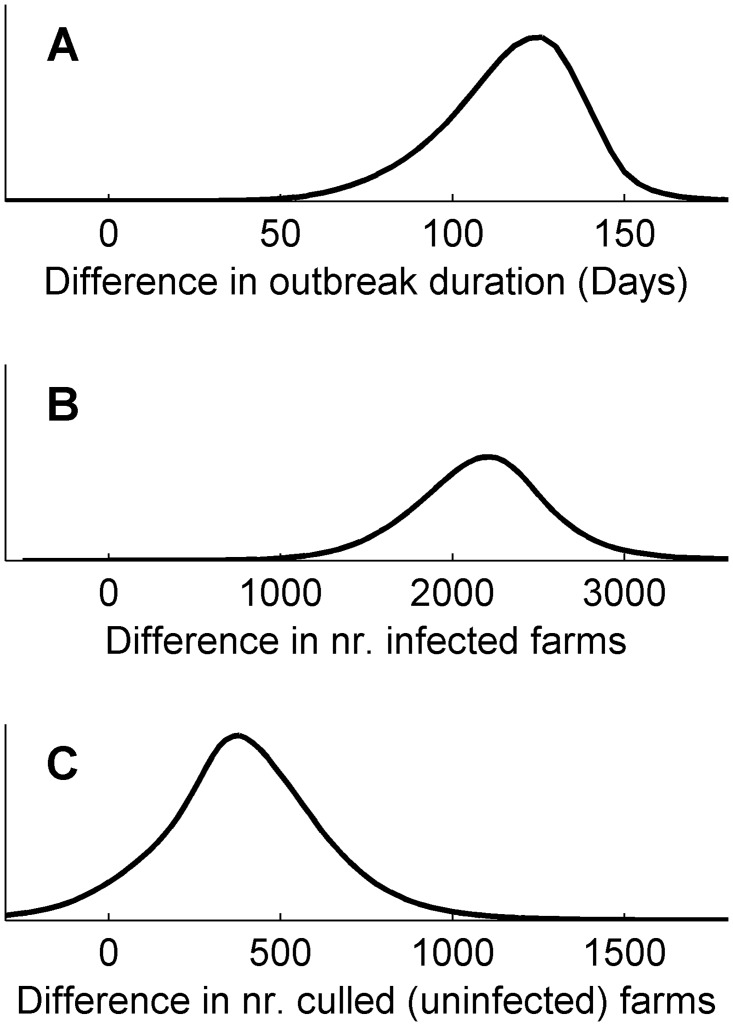
Posterior estimates of difference between controls. The figure shows the marginal posterior predictive estimates of difference in outbreak duration (Panel A), number of infected farms (Panel B) and number of control culls (Panel C) between the implemented and alternative control action. The figure shows the result of the large discrepancy ensemble.

The proposed multi-quantity method can be implemented with either NHW, SHW or IHW schemes. Here we aim to illustrate the effect of using multiple quantities and focus on the SHW scheme. Fig 6 plots the marginal posterior density of mean outbreak duration under the two control actions as estimated for the multiple quantity analysis (solid) together with the corresponding estimates for the single quantity analysis (dashed). The figure illustrates how inclusion of multiple quantities in the analysis leads to tighter distributions, centered on projections for *i =* 1. The multi-quantity analysis produces a probability distribution of all considered quantities, and Fig 7 further illustrates how the marginal posterior densities are located above zero for all three considered quantities.

**Fig 8:**
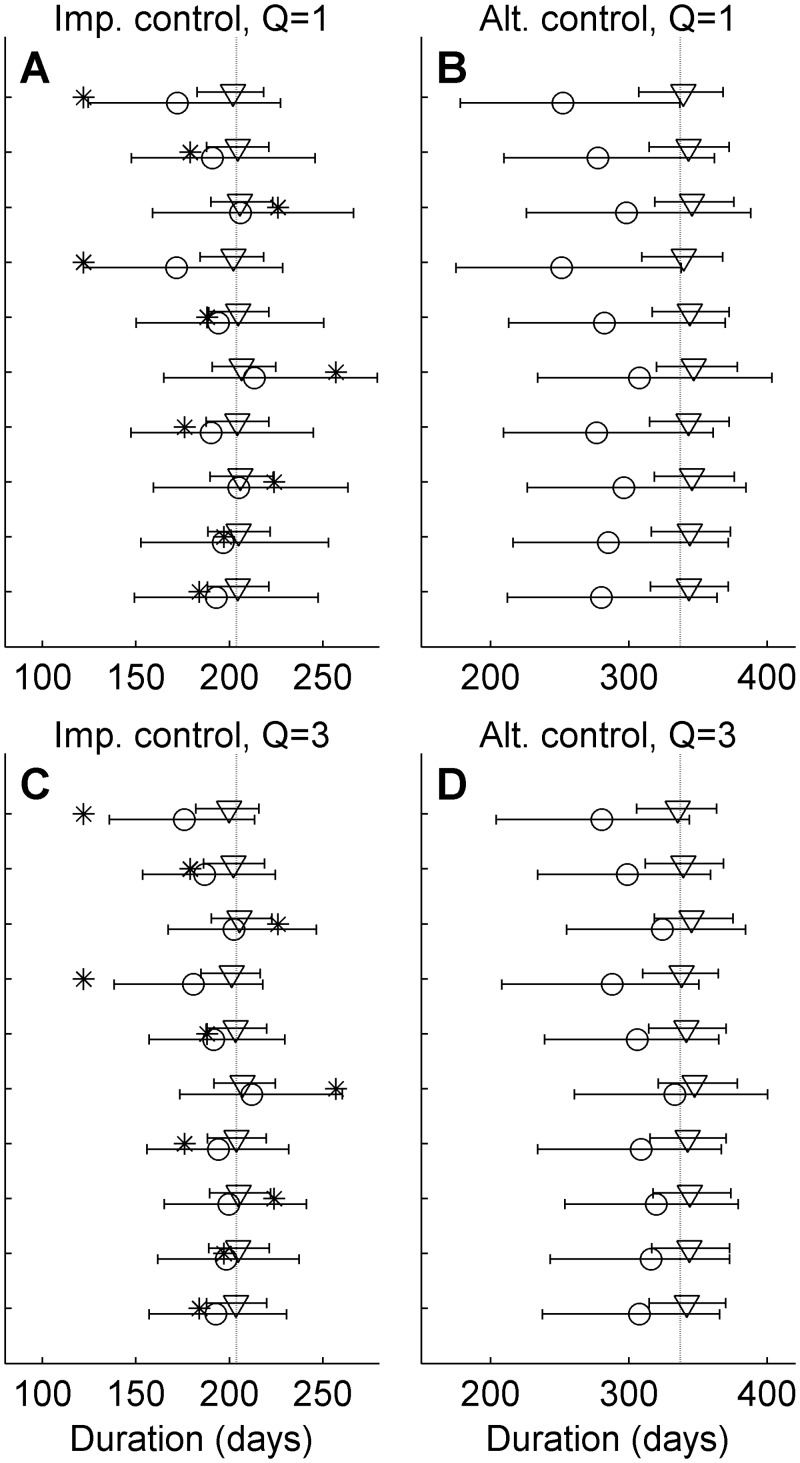
Analysis of synthetic data. Triangles and circles indicate the median posterior estimates of mean outbreak duration after analysis with the small and large discrepancy ensemble, respectively, under the implemented (Panels A and C) and alternative (Panels B and D) control action. Results are shown for ten realizations of synthetic data simulated with *k1* = *k2* = 1. The error bars indicate the 95% credibility interval, the dashed lines the true values and the star the individual realization (expected to frequently lie outside the predicted mean). Panels A and B show the results of single quantity analysis (only outbreak duration) and Panels C and D the corresponding results of analyses where number of infected farms and control culls are included.

To illustrate the performance of the method under different conditions, we also analyzed simulated outbreaks. Fig 8 shows the posteriors of mean duration for outbreaks simulated with the *k*_1_ = *k*_2_ = 1 parameterization and applying the small (triangles) and large (circles) discrepancy ensembles, represented by the median values and error bars indicating the 95% central credibility interval. Note that individual realizations, indicated by stars, are expected to frequently be outside of the credibility envelopes. Error bars are inclusive of the true mean outbreak duration (dashed lines) for all ten analyzed realizations for both the implemented and alternative control actions. However, the credibility envelopes are tighter and medians closer to the true value for the multi-quantity analysis. This indicates that the ensemble prediction is improved by including multiple quantities.

**Fig 9:**
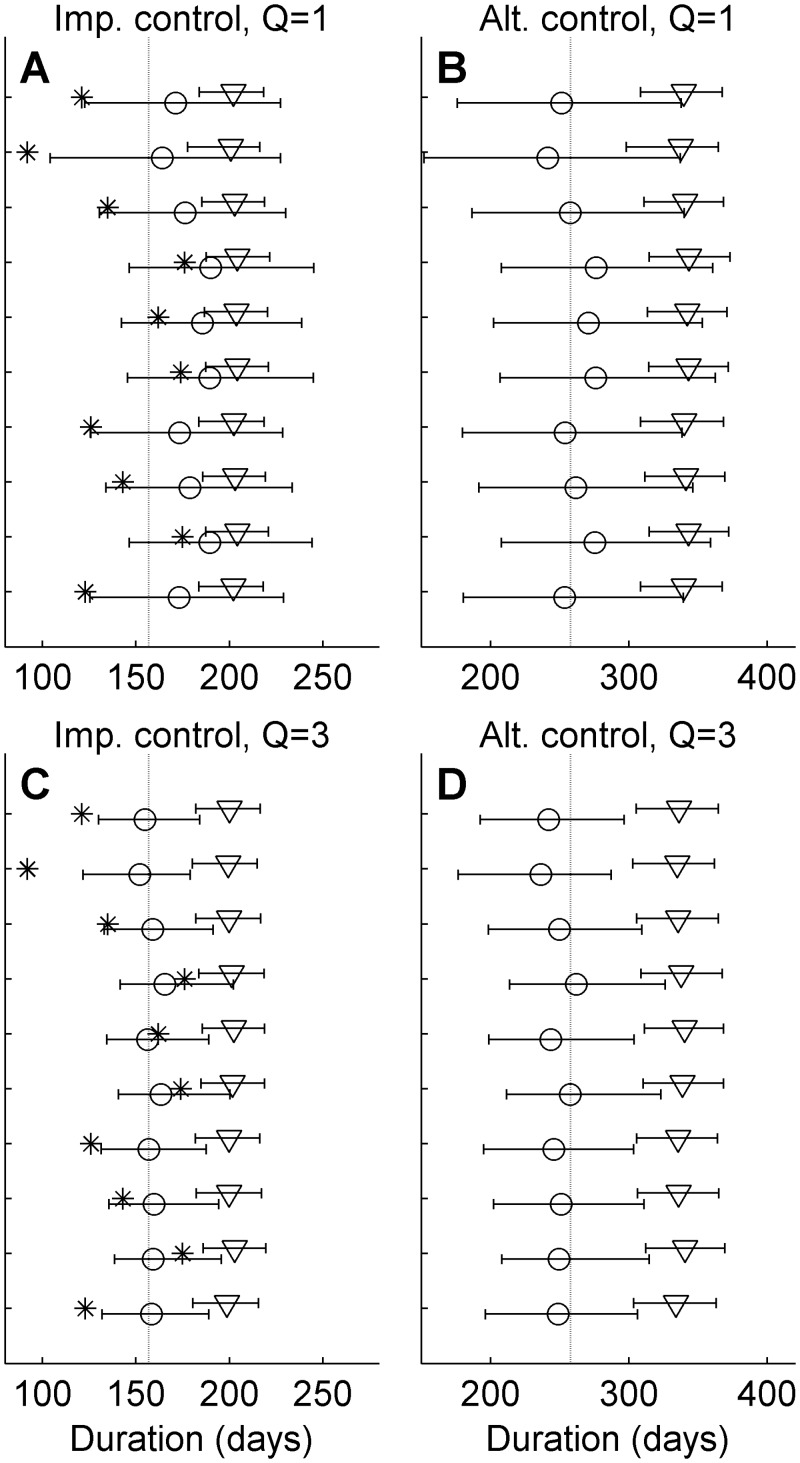
Analysis of synthetic data. Triangles and circles indicate the median posterior estimates of mean outbreak duration after analysis with the small and large discrepancy ensemble, respectively, under the implemented (Panels A and C) and alternative (Panels B and D) control action. Results are shown for ten realizations of synthetic data simulated with *k1* = *k2* = 0.9. The error bars indicate the 95% credibility interval, the dashed lines the true values and the star the individual realization analyzed (expected to often lie outside the predicted mean). Panels A and B show the results of single quantity analysis (only outbreak duration) and Panels C and D the corresponding results of analyses where number of infected farms and control culls are included.

When applying the analysis to outbreaks simulated with the *k*_1_ = *k*_2_ = 0.9 parameterization (Fig 9), the large discrepancy ensemble error bars are still consistently inclusive of the true value. As with Fig 8, credibility envelopes are tighter for the multi-quantity analysis. The error bars of the small discrepancy ensemble that all rely on simulations with parameterizations with higher *k*_1_ and *k*_2_ than the true value, are not inclusive of the true value, indicating that the small discrepancy ensemble fails in predicting the true values of the outbreak.

## Discussion

Ensemble modeling is appealing because it offers the possibility to combine multiple projections. Within weather forecasting, the approach has given more robust predictions, and we could expect that to be the case for epidemiology as well. However, there is a need for the development of methods describing how to combine several epidemiological projections. The aim of this study was to investigate the possibility of using the Bayesian framework introduced by Tebaldi et al. [47]. We find that it is a promising approach, for primarily three reasons.

Firstly, when the methodology is implemented in a hierarchical Bayesian framework, it provides an appealing interpretation of model exchangeability. Essentially, projections and their underlying modeling assumptions are treated as random draws from a population of possible projections. By estimating the hierarchical parameters *a*_*λ*_ and *m*_*λ*_ jointly with individual precisions (weights) *λ*_*i*_, the characteristics of this hypothetical population are estimated. Smith et al. [61] used a similar approach for climate ensembles and pointed out that this reduces the impact of which models are included or excluded in the ensemble. That is, we should expect to get similar results when using a different set of model assumptions if they are chosen independently. We stress that this interpretation is more valid for multi-model ensembles, however. Also, the term “random draws” should not be interpreted as arbitrary. Rather, the interpretation is that models should come from a population of well-informed, reasonable models. The analysis treats the outputs of the performed simulations under different assumptions as data (Eq 1, Table 1), and as such they are used to inform the quantities of interest (*μ* and *v*). This may seem counterintuitive, yet it only serves as a formal means to combine the results of multiple projections, and by Eqs (7) and (20), these are combined with available outbreak data.

Secondly, the framework can handle several different weighting schemes simultaneously. The original methods introduced by Tebaldi et al. [47] used convergence and bias to assess weights. Here, we further extend the framework such that informative priors can be included to inform the weights, thus relaxing the supposition that all modeling assumptions are a priori exchangeable. Epidemiological predictions suffer from lack of available data to assess model bias, and we propose that expert opinions will play a larger role than in other fields of research. With the analytical tool proposed here, a policymaker can choose to include a range of projections based on different modeling assumptions, yet give them different weights, rather than including one or a few (given a weight of one) and excluding others (given a weight of zero). When using different mechanistic models, subjective trust in the different models can be incorporated by using methods of prior elicitation based on expert opinion [38]. Importantly, our methods can incorporate these subjective beliefs in the hierarchical framework, requiring only the specification of the a priori relative confidence in the underlying assumptions of the projections. Definition of an individual, fixed prior would undoubtedly be cumbersome to elicit from expert opinion; it would not be feasible to ask policymakers to define an individual gamma prior for each modeling assumption.

Here we used ensembles based on projections of the same model with different parameterizations, demonstrating the possibility to explore parameter space, yet with unequal probabilities of different parameterizations. Uncertainty about parameters will be an issue for most epidemiological models, and we propose that multi-model ensembles should incorporate projections with different models and different parameterizations. Thus, different mechanistic assumptions as well as parameter uncertainty would be incorporated in the ensemble.

Thirdly, the framework produces easily interpretable probability distributions. It is important that communication with policymakers include uncertainties about prediction rather than just the most likely outcome [16]. In the ensemble context, these uncertainties take into account different assumptions about the transmission process. Gårdmark et al. [32] suggested that uncertainty should be communicated with policymakers by presenting the full range of predicted outcomes. However, that would give equal weights to all included projections and would require that the results be communicated with a detailed description of all assumptions made, thus allowing the policymaker to decide how much to trust each modeling assumption. This would be a cumbersome task, particularly for detailed simulation models that rely on a large number of parameters. We therefore argue that it is beneficial to communicate the aggregated and weighted result as easily interpretable probability distributions. With further modifications of the methodology, we propose that the approach could also be used as a forecasting tool during an outbreak, e.g. by letting *x*_*i*_ and *y*_*i*_ denote current and future numbers of infected farms. In such a situation, there is a great need for rapid and clear communication of model results to aid policy decisions. The visual manner in which uncertainty is presented using probability distributions makes them easy to understand and communicate [62].

We here show that these distributions are sensitive to the choice of priors when using the NHW method (Fig 2, panels A-D, Fig 3, panels A, B). However, the impact of the prior is heavily reduced when using the hierarchical framework (Fig 2, panels E-H, Fig 3, panels C, D). Thus, our results demonstrate that the hierarchical approach is preferred for ensemble modeling and using the non-hierarchical approach can lead to spurious conclusions. We argue that this would also be the case for other fields, such as climate ensembles, but it is likely to be a larger concern for epidemiology where data to modify the prior are fewer. Considering Eq (11), we could ensure that *b* has little contribution to the denominator if *τ*_0_≫λ_i_ for all *i*, ensuring that the prior has little contribution to the posterior. For climate considerations, we envisage that the precision of natural variability, *τ*_*0*_, would be large relative to each *λ*_*i*_ if bias is assessed by comparing model simulations to long time series of climate data. For epidemiological considerations, this would however rarely be the case. In the proposed method, we instead inform *τ*_*0*_ largely by the simulation outputs, letting the projections of the ensemble determine how variable outcomes are.

Climate modeling, from which the proposed method is adapted, is primarily concerned with differences between current and future mean climate variables [24]. Epidemiology is not only concerned with mean projections but also with other quantities such as the probability of very large or long outbreaks occurring. Fig 2, panels G and H illustrates the probability of a given epidemic duration occurring for a single outbreak under the two control actions with the preferred SHW method. Comparing the posterior predictive distribution to the density of merely lumping the results of all simulations, as illustrated by the colored bars, the posterior predictive distribution of the ensemble method has a lower probability of both very long and short outbreaks. This is because projections of such outbreaks are down-weighted when their bias is assessed in the analysis; the observed outbreak duration would be unlikely under the modeling assumptions that produce these projections. Thus, ensemble methods that give equal weights to all projections can overestimate the uncertainty about outbreaks, preventing the models from informing appropriate policy decisions.

We have further extended the methodology to allow for informative priors on the weights. Compared to climate models, epidemiology often has far less available data to assess model bias. As such, expert opinion will often play a larger role within this field. Fig 4 illustrates the behavior of the ensemble prediction under such informative priors. When up-weighting projections for *i =* 1, which is also likely under the observed outbreak duration, the posteriors are shifted towards these projections and produce tighter distributions. This is also found when up-weighting the outlier, *i* = 5, in the small discrepancy ensemble (Fig 4, panels A and B), in which no projection is particularly unlikely for the observed duration. Projection *x*_5_ is however unlikely in the large discrepancy ensemble. As a result, the effect of up-weighting the underlying modeling assumptions of this projection primarily makes the distribution wider, resulting from a larger uncertainty about individual weights (Fig 5). This is to be interpreted such that if expert opinions a priori determine that a modeling assumption that is unlikely to predict the observed data is better than other assumptions, the conclusions should be that there is less information in the ensemble as whole. However, when expert opinions are well informed and do not contradict with observed data, they can lead to more precise predictions.

It should be stressed that discrepancy among projections in the ensembles should be viewed as relative to *τ*_*0*_, the estimated variability in outbreak duration given the initial conditions. A crucial difference between the original method applied to climate change and the epidemiological consideration presented here is that *τ*_*0*_ is unknown for the latter and therefore needs to be estimated. We argue that in the absence of multiple outbreaks, it is sensible to inform this by the model simulations. Stochastic simulations are often used to estimate the range of outcomes for non-ensemble projections [1,17,18,23], and we propose that when extending the use of models to the ensemble context, they can be used to estimate this feature as well. We have therefore chosen a Bayesian model structure where *τ*_*0*_ is informed largely by the within projection variability, τ_1_, τ_2_,…, τ_n_, via the Gamma(*a*_*τ*_, *b*_*τ*_) distribution in Eq (8). All projections of the implemented control action contribute equally to this distribution in the method presented here, thus we are giving equal weights to all modeling assumptions in terms of informing *τ*_*0*_. Estimation of different weights in terms of informing *τ*_*0*_ based on a single outbreak, analogous to the estimation of ***λ***, would not be conceivable. However, if policymakers believe that some modeling assumptions are more reliable in terms of capturing the variability of outcomes, we envisage that the Bayesian model structure can be altered to include this. If applied to endemic disease, *τ*_0_ could be informed similarly to the natural variability of temperature in climate application, and the algorithm we supply is set up to handle this situation. Also, data from multiple outbreaks could be used to inform *τ*_*0*_ when available. Yet, data quality will rarely be comparable to climate data, which highlights one of the major challenges for epidemiological modeling.

We also provide a multi-quantity extension of the Bayesian ensemble framework. Fig 5 shows that when adding number of infected and culled farms to the analysis, the marginal posteriors of outbreak duration become narrower and centered on *x*_*1*_ and *y*_*1*_, i.e. the projections based on the most likely scenario. This illustrates that predictions can be improved by incorporating multiple quantities when assessing the weights.

The main scope of this study is to introduce ensemble methods to the field of epidemiology rather than to produce inference about the 2001 FMD outbreak. However, Fig 7 illustrates the types of conclusions the method can provide. The three quantities we include in the multi-quantity analysis are all of great concern to policy makers when assessing the impact of control actions. The probability distributions represent the ensemble predicted difference in the outcome of the outbreak if the control action had excluded culling of CPs. The distributions all have most of the density above zeros, indicating that excluding culling of CPs would most likely have resulted in a prolonged and larger outbreak. We should however point out that these results are based on a single model. To make more robust predictions, we propose that the same type of analysis be made with projections of different models.

We also analyzed simulated data to provide a more general depiction of the performance of the method under different conditions. Fig 8 shows the result of analysis of ten simulated outbreaks with the parameterization in the center of the ensemble, i.e. *k*_1_ = *k*_2_ = 1. As this is in agreement with both the small and large discrepancy ensemble, the true values (dashed lines) consistently lie within the 95% credibility intervals. However, when using the *k*_1_ = *k*_2_ = 0.9 parameterization, the assumptions of the model used to simulate the outbreak is only inclusive of the large discrepancy ensemble, and consequently only the large discrepancy ensemble error bars are inclusive of the true values. Noting that we primarily use the different parameterization as a proxy for different models, this simple simulation example illustrates some obvious but essential points. Ensemble modeling should not be interpreted as a remedy for models based on poor assumptions about the modeled process. It offers the ability to combine multiple assumptions, thus integrating uncertainty with regards to this in the predictions. However, if all models are based on similar but inaccurate assumptions, ensemble modeling will not improve predictions. Intentionally making models similar to each other increases this risk and should be avoided if the models are to be used for ensemble purposes.

Accepting these limitations, we argue that the ensemble approach will be beneficial to epidemiological risk assessment because rather than choosing a single model for the purpose, it offers the possibility to combine projections from models that make mechanistically different assumptions about the transmission process. Thus, uncertainty with regard to this is incorporated in the predictions, which is important as projections of different models have been reported to deviate [63–65]. The use of multi-model ensembles would rely on collaboration of modeling teams, as well as overcoming confidentiality constraints in accessing outbreak data and population demographics. The current development in FMD modeling is seeing encouraging development in that area. The Quadrilateral Epiteam [19] has compared simulation of several outbreak scenarios in a subset of the UK demographics with five different models: NAADSM [45], Netherlands CVI [66], InterSpreadPlus [46], AusSpread [44] and ExoDis [67].

This demonstrates that potential obstacles for multi-model ensembles can be overcome and we envisage that epidemiology will see a shift towards multi-model ensembles to inform policy decisions, as has been seen in climate research [24,25] and weather forecasting [26,27]. Combining the results of multiple models however requires means of weighting these. We conclude that the presented framework is a promising approach because it provides easily interpretable probability distributions of quantities of interest. It also offers an appealing interpretation of model exchangeability, while at the same time combining several different weighting schemes, including a priori beliefs when such are available.

In this study, we introduced this framework by applying it to a simple question: how would exclusion of contiguous premises culling from the control action have affected the outcome of the UK 2001 outbreak? The aim of the study has been to introduce the methodological framework to epidemiology and solve some key issues associated with this transfer, including prior sensitivity, informing weights by expert opinion, using models to inform the variability in the outcome of individual outbreaks and extension to consider multiple epidemic quantities. We have purposely chosen the simple example because it allows for a straightforward transfer from the original climate implementation, and at the same time lets us demonstrate essential concepts and the potential of the framework. Models are however used to answer a range of different questions in epidemiology, and combining multiple projections has the potential to improve the way models are used to inform policy. We argue that the framework we introduce here has great potential, and foresee that many of the questions addressed in epidemiological modeling would require further developments of the Bayesian model, structured to fit with the specific problem. To facilitate this, we have supplied the algorithm (S1 File) and hope that it will aid further development of ensemble methods for epidemiology.

## Supporting Information

S1 File MCMC code written in MatLab. (ZIP)
